# Multi-modal treatment with peptide vaccine, metronomic cyclophosphamide and anti-PD1 monoclonal antibody provides effective control of tumors in multiple models

**DOI:** 10.1186/2051-1426-2-S3-P130

**Published:** 2014-11-06

**Authors:** Genevieve Weir, Olga Hrytsenko, Marianne Stanford, Neil Berinstein, Robert Liwski, Mohan Karkada, Marc Mansour

**Affiliations:** 1 Immunovaccine Inc, Halifax, Nova Scotia, Canada; 2 Ontario Institute for Cancer Research, Toronto, Ontario, Canada; 3 Queen Elizabeth II Health Sciences, Halifax, Nova Scotia, Canada

## 

Future cancer immunotherapies will combine multiple treatments to improve immune responses to cancer through synergistic, multi-modal mechanisms. In a Phase I clinical trial, we found that the immune response to a peptide vaccine targeting survivin, DPX-Survivac, by ovarian cancer patients can be improved by combination therapy with metronomic cyclophosphamide (mCPA). Pre-clinical studies in mice to further understand the mechanisms of this enhanced effect demonstrated that this improvement could be attributed to selective enrichment of antigen-specific CD8+ T cells resulting in improved immune responses detected by IFN-γ ELISPOT, *in vivo *cytotoxicity assay and improved protection from tumors. Combination therapy was also associated with reduced accumulation of myeloid-derived suppressive cells. Efficacy of the vaccine and mCPA combination was limited in mice with advanced tumors, yet antigen-specific CD8+ T cells could be detected within the tumor microenvironment by flow cytometry along with increased expression of PD-1 by qPCR, suggesting that the combination therapy was able to generate strong cytotoxic T cell response but was still subject to tumor induced suppression within the tumor microenvironment. Therefore, we tested whether anti-PD1 therapy could improve the efficacy of vaccine and mCPA combination therapy in advanced tumor models. Using HPV16E7 expressing C3 tumor model, we found that mice bearing advanced tumors treated with the tri-therapy consisting of cancer vaccine, mCPA and anti-PD1 could more effectively eradicate tumors than those treated with single or double therapy combinations. The tri-therapy combination was also effective in treating other difficult to treat tumor models such as B16-F10. These results highlight the usefulness of pre-clinical mouse models in evaluating the mechanisms through which multiple immune therapies can co-operate. Future work will investigate the potential of adding anti-PD1 treatment to clinical trials evaluating DPX-Survivac in combination with mCPA.

